# The effects of video game therapy on balance and attention in chronic ambulatory traumatic brain injury: an exploratory study

**DOI:** 10.1186/s12883-017-0871-9

**Published:** 2017-05-10

**Authors:** Sofia Straudi, Giacomo Severini, Amira Sabbagh Charabati, Claudia Pavarelli, Giulia Gamberini, Anna Scotti, Nino Basaglia

**Affiliations:** 1 Neuroscience and Rehabilitation Department, Ferrara University Hospital, Ferrara, Italy; 2 School of Electrical and Electronic Engineering, University College Dublin, Dublin, Ireland

**Keywords:** Gaming, Traumatic brain injury, Balance, Attention deficit, Mobility

## Abstract

**Background:**

Patients with traumatic brain injury often have balance and attentive disorders. Video game therapy (VGT) has been proposed as a new intervention to improve mobility and attention through a reward-learning approach. In this pilot randomized, controlled trial, we tested the effects of VGT, compared with a balance platform therapy (BPT), on balance, mobility and selective attention in chronic traumatic brain injury patients.

**Methods:**

We enrolled chronic traumatic brain injury patients (*n* = 21) that randomly received VGT or BPT for 3 sessions per week for 6 weeks. The clinical outcome measures included: i) the Community Balance & Mobility Scale (CB&M); ii) the Unified Balance Scale (UBS); iii) the Timed Up and Go test (TUG); iv) static balance and v) selective visual attention evaluation (Go/Nogo task).

**Results:**

Both groups improved in CB&M scores, but only the VGT group increased on the UBS and TUG with a between-group significance (*p* < 0.05). Selective attention improved significantly in the VGT group (*p* < 0.01).

**Conclusions:**

Video game therapy is an option for the management of chronic traumatic brain injury patients to ameliorate balance and attention deficits.

**Trial registration:**

NCT01883830, April 5 2013.

## Background

Postural instability, due to failures in the complex interactions between the sensory, motor and musculoskeletal systems, is very common in traumatic brain injury (TBI) and persists in one third of survivors years after the trauma [1]. Balance impairment can limit the activities of daily living and active participation in a social life. Similarly, attention deficits are TBI sequelae that affect 39–62% of TBI survivors [2] and might interfere with a person’s ability to safely complete motor tasks and learn new activities [3]. Selective attention is pivotal for everyday life to enhance the stimuli that are relevant and suppress the representation of stimuli that are distracting. Thus far, balance outcomes have been tested using different modalities [4–9]. Balance and postural stability improved after conventional physiotherapy based on motor learning principles specifically tailored for treating postural and coordination dysfunctions in an open trial performed with patients with mild-to-moderate TBI [4]. Focusing on specific gait therapies, body weight-support training on a treadmill (BWSTT) was not found to be superior to overground walking [5] or different robotic devices [6]. Other approaches that have been explored include the use of a biofeedback device to improve perception of external perturbations [7], vestibular rehabilitation [8] or a combination of cerebellar intermittent theta burst stimulation (iTBS) and physiotherapy [9]. Nevertheless, this state-of-the-art work in balance rehabilitation in TBI cannot be translated into useful evidence-based recommendations, and the use of new interventions such as virtual reality (VR) has been encouraged [10]. In recent years, VR technologies have begun to be used as a treatment tool in rehabilitation given their low-cost, high portability, off-the-shelf nature and ability to deliver engaging, high-repetitive, task-oriented, standardized, active learning therapies [11, 12]. Moreover, VR-based rehabilitation typically provides augmented feedback during training that can contribute to learning motor skills [13]. Virtual reality also increases patient attention and motivation, which are essential components of learning [14]. Indeed, it has been hypothesized that VR efficacy relies on virtual reward-based learning through a dopaminergic facilitation of cortical and subcortical networks [15]. Motivation and rewards can affect attentional processes in healthy subjects [16, 17] and patients with hemispatial neglect [18]. More recently, gaming consoles (e.g., Nintendo Wii, Xbox Kinect) have been introduced in clinical and research settings as a low-cost means of delivering VR training. With Xbox Kinect, patients can see their movement in real time, and the feedback results are more accurate and realistic compared with other devices with external controllers. Moreover, gaming therapy can be delivered at home, promoting self-management strategies to improve motor function and long-term outcomes [19].

There is limited evidence supporting the use of VR rehabilitation on balance and mobility in TBI survivors [20]. The first attempts were made by Sveistrup et al. [21] and Thornton et al. [22]. These authors used the IREX system for balance training. Sveistrup et al. found balance improvements both in VR and conventional exercise groups [21], and Thornton et al. reported greater enthusiasm after VR therapy by TBI patients and caregivers compared with controls [22]. In the past few years, video game therapy (VGT) has been tested in subacute TBI patients undergoing multidisciplinary rehabilitation with positive effects on balance [23] and in chronic TBI patients using customized games [24]. However, the aforementioned studies did not explore the hypothesis that VGT would ameliorate attention through reward-based learning, in addition to motor function. The aims of this exploratory study were to test the effects of a commercially available VGT on balance and selective attention in ambulatory chronic TBI patients compared to a standardized balance platform training (BPT). We hypothesized that the VGT, and in particular “action video games,” would improve selective visual attention more so than BPT. In a previous study, video games were shown to improve a patient’s ability to focus on a target and to ignore distracting information not present in BPT [25]. In action video games, players constantly receive feedback about the accuracy of their predictions, which is a fundamental step in engaging the reward system [26]. Video game therapy can improve executive attention components such as control of the automatic response, control of goal-directed behavior and the ability to inhibit irrelevant stimuli [25]. Those cognitive components are part of a “top-down” attentional control mechanism that directs attention in a controlled manner that depends on our personal goals and expectations. Our hypothesis is that VGT would activate those cognitive components of learning more so than BPT, leading to an improvement in balance and attention in a convenience sample of TBI patients.

## Methods

This exploratory, randomized, controlled study (NCT01883830; April 5 2013) was approved by the Ferrara University Hospital Ethics Committee (Ferrara, Italy), and all subjects signed a consent form prior to participating in any procedures. The subjects were enrolled from patients discharged at home (former patients included in the clinic database) or those patients receiving multidisciplinary inpatient rehabilitation at Ferrara University Hospital. During the multidisciplinary rehabilitation, physical, cognitive, behavioral and vocational therapy were delivered according to the abilities and needs of each patient. However, to reduce possible confounding effects on our measures (balance and selective attention), no additional specific training, except for the research study, was administered for balance and attention. The inclusion criteria included: (i) an age between 18 and 70 years; (ii) a diagnosis of chronic TBI (>12 months); (iii) a balance deficit identified by a Community Balance & Mobility Scale (CB&M) score < 65. The exclusion criteria included: (i) the presence of other neurological diseases; (ii) severe cognitive Levels of Cognitive Functioning (LCF) < 6 or behavioral disorders; (iii) reliance on the use of walking aids. Patients were randomized according to block randomization and allocated into two groups: VGT or BPT. Each patient received three 1-h sessions per week over the course of 6 weeks.

### Video game therapy

Video game therapy was delivered with a video game console (X-Box 360 Kinect, Microsoft, Inc., Redmond, WA). Pre-selected games were chosen from “Kinect Adventures” and “Kinect Sports” that encompassed a wide range of motor activities in a standing position. Specifically, balance and mobility-related motor tasks, such as side stepping, lateral weight shifting, jumping, walking (lateral, forward and backward) and arm goal reaching were trained. During the first session, a list of games was tested according to the patients’ characteristics, desires and functional level. In the following sessions, games were proposed with a block practice approach. Within each game, progression proceeded over time according to the patients’ abilities and successes. Video game therapy provided different types of feedback: visual and augmented (knowledge of both results and performance). Patients exercised for 2–5 min during each game with a rest period if necessary. During the sessions, the patients were carefully supervised by a physiotherapist who monitored the safety of the patients (e.g., risk of falls, impulsive reactions) and provided external feedback.

### Balance platform therapy

Balance/rebalancing, postural stability and weight-shifting exercises with and without visual feedback were administered using a balance platform (Biodex Medical Systems, Inc., Shirley, NY) that had been tested previously in multiple sclerosis patients [27]. Each task was trained for about 2 min, and the patients were provided with a rest period between the tasks if necessary. During the first session, the tasks were performed at an “entry level,” and the exercise progression was adjusted over time according to the patients’ functional level (intermediate and difficult level). Balance platform therapy offered visual feedback and knowledge of performance (augmented feedback). The physiotherapist, as during VGT, provided additional external feedback.

### Outcome measures

Clinical, posturographic and cognitive tests were assessed pre- and post-treatment. We selected balance measures that explored a broad range of motor tasks (both static and dynamic), were suitable for assessing ambulatory patients and were less susceptible to a ceiling effect [28–30]. We assessed balance and mobility using the CB&M. This 13-item scale measures challenging motor tasks necessary for mobility in the community. The tasks have components of speed, precision and accuracy such as tandem walking, running, walking while looking laterally, backward walking and descending stairs [28, 29]. Furthermore, we administered the Unified Balance Scale (UBS) to assess each patient’s ability to maintain his or her balance, either statically or while performing functional movement. This 27-item scale derives from three well-established balance scales (Berg Balance Scale, Tinetti Scales and Fullerton Advanced Balance Scale) that address five balance domains: quite stance, anticipatory postural adjustments, sensory orientation, external perturbations and stability in gait [30]. We also administered the Timed Up and Go (TUG) test, which measures mobility. We gave patients verbal instructions to stand up from a chair, walk 3 m, cross a line marked on the floor, turn around, walk back and sit down [31].

### Selective visual attention (Go/Nogo task)

The Go/Nogo task was taken from a German standard battery used to test attentional functions [32]. This task consists of five types of stimuli: two of them are target stimuli in which the patients were required to press a button, as quickly as possible, if one of the two defined targets is presented. This test measures the selective attention as the time of reaction and the impulsivity as the number of false alarms (a button press when the patient viewed a non-target stimulus).

### Static balance

In this test, patients were asked to step on the central region of the force plate, always facing in the same direction, and to assume an up-right posture with their arms lying alongside their legs and the lateral malleoli distance equal to the iliac spine distance. The patients were asked to either keep their eyes open (looking straight ahead at a 3 m distant visual reference) or closed. For each condition, three 90-s trial were recorded, and we allowed a 5-min break between trials. The eyes opened (EO) condition reflected a highly automatic activity, and the eyes closed (EC) condition detected sensory integration deficits [33]. The *x* and *y* positions of the center of pressure (COP) of the subjects were calculated from forces and moments measured by the force platform. Parameters related to postural sway and balance were calculated from the COP trajectory during each trial, namely the anteroposterior (AP), mediolateral (ML) and total path lengths and the sway speed.

### Statistical analysis

We compared baseline characteristics between the groups to assess the quality of randomization. Pre-post effects within groups were investigated using the Wilcoxon matched-pairs signed-rank test, and between-groups differences were explored using the Wilcoxon rank-sum test. Statistical analysis was performed using STATA 13.1 software (College Station, TX: StataCorp LP). Significance was recognized for *p* < 0.05.

## Results

**Fig. 1:**
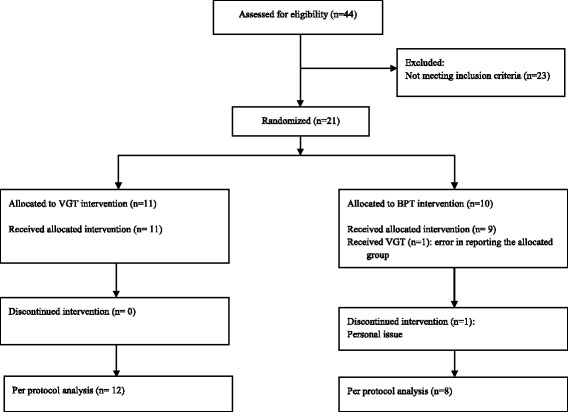
The study CONSORT flow diagram

We enrolled 21 ambulatory chronic TBI patients (17 males, 4 females) with a median age of 36 (12 IQR) years; one patient dropped out for personal reasons. The cohort’s median duration since TBI was 4 years (7 IQR). The study flow diagram is shown in Fig. 1.

The clinical and demographic characteristics of the patients are summarized in Table 1. The two groups were similar in demographic and clinical characteristics except for time since TBI (*p* = 0.02).

**Table 1:** Sample characteristics (reported as median and IQR)

	VGT	BPT	All	*P* value*
Age (years)	30.5 (16)	37 (10)	36 (12)	0.14
Sex (M/F)	10/2	7/2	17/4	0.74
TBI onset (years)	2 (6)	8 (16)	4 (7)	0.02
Inpatient rehabilitation/discharged at home	4/8	2/7	6/15	0.57

*VGT* video game therapy, *BPT* balance platform therapy, *TBI* traumatic brain injury; *Wilcoxon rank-sum test or Pearson’s chi-squared

### Balance and mobility clinical tests

For the CB&M, we found a significant treatment effect in both groups. Conversely, TUG and UBS outcomes improved only in the VGT group. Between-group differences were highlighted with respect to TUG and UBS improvements (*p* < 0.05).

### Static balance

No significant effects after training in AP, ML and total path length or sway speed were found. However, a trend of improvement in the EO condition was noted in the VGT group.

### Go/Nogo task

Selective attention improved significantly in VGT group (*p* < 0.01). Impulsivity was reduced after both treatments (−0.6 ± 1.2 false answers in the VGT group and −0.5 ± 1.5 false answers in the BPT group) but not significantly.

The results are summarized in Table 2.

**Table 2:** Results (reported as median and IQR)

	VGT (*n* = 12)				BPT (*n* = 8)			
	Pre-treatment		Post-treatment		Pre-treatment		Post-treatment	
Clinical tests								
UBS	43 (20.5)		49.5 (20.5)**^∞^		49 (18.5)		51 (20.5)	
CB&M	17 (15)		25 (15.5)**		25 (32)		25.5 (31.5)*	
TUG (s)	18.7 (16.1)		16.4 (9.4)**^∞^		14.0 (20.3)		15.4 (16.2)	
Force platform	EO	EC	EO	EC	EO	EC	EO	EC
ML path length (mm)	154.9 (56.0)	161.2 (68.3)	140.7 (83.9)	188.1 (85.0)	169.5 (539.5)	218.8 (508.3)	201.0 (128.3)	233.5 (145.8)
AP path length (mm)	223.7 (80.9)	312.0 (141.1)	171.2 (137.6)	311.3 (147.9)	258.3 (127.6)	332.5 (419.6)	262.7 (226.1)	321.6 (480.4)
Sway speed (mm/s)	15.6 (6.9)	19.2 (4.3)	12.7 (8.6)	19.7 (10.1)	18.2 (24.4)	22.9 (35.8)	20.9 (9.8)	23.5 (22.8)
Tot path length (mm)	309.5 (137.0)	382.0 (85.6)	252.1 (170.7)	392.0 (201.6)	362.0 (486.4)	456.3 (714.3)	416.3 (194.8)	468.5 (454.5)
Go/Nogo task								
reaction time (ms)	569.5 (205)		557 (179)**		568 (146)		576 (166)	
False answers (n)	0 (2)		0 (0)		0 (0.5)		0 (0.5)	

*VGT* video game therapy, *BPT* balance platform therapy, *UBS* Unified Balance Scale, *CB&M* Community Balance & Mobility Scale, *TUG* Timed Up and Go, *ML* mediolateral, *AP* anteroposterior; *p < 0.05; **p < 0.01 Wilcoxon matched-pairs signed-rank test; ^∞^ *p* < 0.05Wilcoxon rank-sum test

No adverse effects were reported during the training periods.

We performed a sample size calculation using the UBS improvements before and after the treatments. We estimated an effect size of 0.84 (d Cohen). Therefore, 48 patients (24 for group) would be required for a study with a power of 80% and an alpha of 5% (allocation ratio 1:1) in a future study.

## Discussion

This exploratory study is the first to use the Xbox Kinect in chronic ambulatory TBI patients for balance and attention training. Chronic TBI patients are usually discharged to their homes when they reach a functional plateau; they accordingly do not receive any form of rehabilitation, even if postural instability and mobility deficits are often reported [34]. However, our results confirmed previous studies [21, 24, 35] that revealed how even in a chronic phase TBI survivors can improve their mobility and dynamic balance with a therapy based on use-dependent neuroplasticity principles [36].

Our primary findings are that dynamic balance and overall mobility improved after training; moreover, selective attention resulted increased, revealing a significant cognitive engagement during VGT.

We investigated balance using validated clinical tests and posturographic assessments. The CB&M scores, which evaluated each patient’s ability to perform highly challenging balance and mobility tasks, were significantly improved after both treatments. However, only in VGT group were the gains clinically significant (8 vs 0.5 points). This finding is likely due to the fact that VGT trains patients in more challenging and dynamic motor tasks, such as side stepping, reaching high and low, lateral weight shifting and jumping.

Mobility, measured by the TUG test, was significantly increased only in the VGT group. We noted differences in mobility between the groups. Moreover, 58% of patients exceeded the minimally detectable change (MDC) set at 2.9 s [37]. Similarly, the UBS that explored both static and dynamic balance was significantly improved in the VGT group, with differences between the groups. This new balance outcome measure covers all of the relevant aspects of balance, exhibits good psychometric properties and avoids the well-known ceiling effect characteristic of other balance scales [38]. Additionally, the UBS was more suitable for detecting differences among the groups compared with the CB&M.

In terms of postural sway, our sample swayed more in the EC condition, which is consistent with the visual deprivation that underlines these patients’ sensory integration deficits [39]. After VGT, a slight but not statistically significant improvement was noted in the EO condition. The lack of improvement in static balance is consistent with the fact that it is not considered a predictor of mobility in TBI survivors measured as COP displacement in quiet standing [40]. Furthermore, VGT trains complex movements that require more acceleration, coordination and precision than standing tasks. For this reason, VGT, compared with BPT, appears to ameliorate dynamic rather than static balance domains. Examining other trials that use video games in TBI survivors [23, 24], Cutberth et al. found balance improvements after subacute TBI patients trained with a Nintendo Wii Fit balance board during multidisciplinary rehabilitation. These authors did not highlight any differences compared with standard therapy, which might be due to the fact that their sample was in a spontaneous phase of recovery or that multiple modes of therapy (VR + inpatient rehabilitation) were delivered [23]. In a subacute phase, it is logical that Nintendo Wii Fit—which permits more active guidance by physiotherapists—is more appropriate. In a chronic phase, training with Xbox Kinect can be introduced. Ustinova et al. proposed a Kinect-based customizable therapy for differing ranges of impairments that vary from mild to moderate in TBI severity [24].

In terms of attention assessment, our findings suggest that VGT can improve selective attention measured with the Go/Nogo task. This result is consistent with previous studies that highlighted how video game feedback is capable of improving visual selective attention in habitual players [41]. Video games increase information processing procedures to provide either an adequate response for stimulus processing (e.g., an increase in visual acuity [42] or contrast sensitivity [43] or to enhance top-down attentional control as an ability to strategically allocate one’s attention [44])﻿. Attentional control implies some skills related to executive functions such as goal-directed behavior, strategic allocation of one’s attention, error monitoring and cognitive flexibility [45]. Game benefits might reflect shifts in strategy rather than changes in more basic cognitive capacities [46]. Our results confirm some previous studies [26] that showed that action video games resulted in different effects in a patient’s selective attention compared with other “non-action” games (strategic or role-playing games). In our study, VGT was associated with a higher perception, higher attentional capture and a higher motor-load than BPT. In addition, video games had an influence on the reward system. Consequently, the involved reward system represents a key step in learning and cognitive processing [47]. Increased attention can help motor skill learning and functional recovery in TBI survivors and can partially explain the functional gains obtained by our cohort of patients who received VGT.

This exploratory study presents several limitations: our finding cannot be generalized to the entire TBI population. Specifically, Xbox Kinect, like other gaming devices, was developed for a healthy population and is not adjustable for people with cognitive and sensory-motor impairments. Furthermore, TBI survivors with extended frontal damage may not benefit from a reward-based learning delivered by VR. Additionally, the therapist was not blinded to the treatments received, which may represent a potential source of bias. Also, the VGT and BPT groups were significantly different at baseline with respect to the time since TBI (2 vs 8 years). We also have to consider that patients with an higher chronicity may have developed more compensatory strategies over time, rendering them less susceptible to modification with the rehabilitative interventions. Finally, five of the 20 patients were receiving multidisciplinary rehabilitation, even in a chronic phase, and the multiple interventions could mask specific effects of VGT or BPT. However, we decided to include chronic TBI patients even if they were undergoing rehabilitation given the preliminary nature of this trial and the difficulty of recruiting members of this particular population.

In future studies, it will be important to evaluate the effect of video games on other attention components (e.g., divided attention) and other executive functions such as working memory and flexibility, which are often impaired in people with TBI [48]. Such an evaluation can help to predict performance after VGT [49], either in the early or later phases of learning. It would also be helpful to use an adaptive video game characterized by a progressive increase in attentional and executive loads in order to make the intervention more effective, even for the most compromised patients.

## Conclusions

Ambulatory chronic TBI patients appeared to benefit from 6 weeks of VGT in terms of dynamic balance, mobility and selective attention. However, these promising results were obtained from a small sample of convenience. Additional studies with more homogeneous and larger samples are required to confirm and better explore the role of video games on motor learning after TBI.

## Abbreviations

AP: Anteroposterior
BPT: Balance platform therapy
CB&M: Community Balance & Mobility Scale
COP: Center of pressure
EC: Eyes closed
EO: Eyes opened
ML: Mediolateral
TBI: Traumatic brain injury
TUG: Timed Up and Go
UBS: Unified balance scale
VGT: Video game therapy
VR: Virtual reality
